# Educational Intervention of Intention Change for Consumption of Junk Food among School Adolescents in Birgunj Metropolitan City, Nepal, Based on Theory of Planned Behaviors

**DOI:** 10.1155/2020/7932324

**Published:** 2020-03-27

**Authors:** Upendra Kumar Singh, Nirmal Gautam, Tulsi Ram Bhandari, Nirmal Sapkota

**Affiliations:** ^1^Department of Medical and Allied Science, Faulty of Public Health, Karnali College of Health Science, Gaushala, Kathmandu 44602, Nepal; ^2^School of Health and Allied Sciences, Faculty of Health Sciences, Pokhara University, Kaski, Pokhara, Nepal

## Abstract

Consumption of junk food among adolescents has been recognized as a serious health problem in the world. Therefore, this study aims to assess the effectiveness of an educational intervention program (interactive lecture) based on the theory of planned behavior (TPB) for reducing junk food consumption among school adolescents in Birgunj Metropolitan City, Nepal. A structured questionnaire was deployed for collecting the data from four government schools. Pretest and Posttest group study design and simple random sampling techniques were used. A multiple linear regression model and a paired *t*-test were used to assess the effectiveness of an educational intervention program. The theory of planned behavior indicates that behavioral intention of junk food consumption was different in pretest and posttest [5.43 ± 1.3 and 7.96 ± 0.3]. Furthermore, the average score of attitude toward junk food consumption was 11.9 ± 1.5 and 16.3 ± 1.6. Meanwhile, perceived behavior control (PBC) toward junk food was also different after intervention [2.42 ± 0.50 and 3.13 ± 0.58]. The interactive lecture method was proved an effective education program for changing the intentions of adolescent students and preventing them from consuming junk food which were statistically significant (<0.05). In addition, behavioral intention of junk food consumption, attitude toward junk food consumption, and perceived behavioral control toward junk food were statistically significant (<0.05). Therefore, study concluded that the intervention program has positive influence on the perceived behavior without control group of school-going adolescents.

## 1. Introduction

Junk food is a form of food which generally contains low nutrients but high fat, saturated fat, sodium, and low fiber [1, 2]. These types of foods show negative health effects (obesity, metabolic disorder, and high cholesterol) in children [3–5]. Even so, consumption of junk food has been increasing by fivefold among adolescents in the last three decades [6–8]. This is due to its good taste, easy availability, affordable cost, choices, and flavor [9]. This increases the consumption of junk food leading to the increase in the risk of obesity and causing public health problem around the world [10, 11].

Globally, it was found that 41 million children and adolescents were the victims of obesity as a result of junk food in 2016 [12]. This effect is seen throughout the world; however, it differs from one country to another. For instance, the consumption of junk food has remained higher in developed countries rather than developing ones, because of the liberalization of trade and foreign investment policy on food and beverage products [8, 12, 13]. In this regard, the change in nutrients found in food has been shown as the burden of obesity [11, 14]. Therefore, considerable investment in intervention strategies should be applied for reducing the consumption of junk food as a goal for any particular nation.

Many researchers, in national and international organizations, have conducted studies based on junk food consumption and have found that there is a linkage between function (physical and cognitive) and proper diet and the body [15–18]. In this regard, proper nutrition facilitates the development of the body and mind among children and adolescents [19, 20]. Additionally, good nutrition during these stages is essential for acquiring skills [21]. Otherwise, it may lead to increased morbidity and mortality among adolescents [22]. It was found that every day, approximately 3,000 adolescents were dying due to preventable illnesses (nutrition deficiency, respiratory infection, and accident) around the world. Moreover, it shows the negative health effects associated with increasing the risk of chronic diseases [23, 24].

In Nepal, changes in food culture have been significant over the past decade. These changes are not only for eating behavior but also for food production and attitude toward it [25]. This is particularly found among the adolescents. Almost one-fourth of the total population is comprised of adolescents [26]. Of them, 54.2%, over half of adolescents have low knowledge of proper food and its consequences; thus, the majority of them were at risk of eating of junk food [27]. In addition, the consumption of junk food varied by age; it was found that the higher proportion of junk food was in early adolescents (93%) as compared to late adolescents (89%) [28]. This difference in the consumption of junk food is determined by the taste and availability and by home and environmental factors [29]. Hence, gaining knowledge of nutrition can be beneficial in improving the health and well-being of adolescents and individuals.

Based on these observations, educational intervention for change in the behavioral intention on the consumption of junk food for school adolescents can help to develop a better understanding of the practice and consumption of proper food. There have been different sites for gathering the information. Meanwhile, this study focuses only on the school setting, which has been identified as an important setting for collecting the information because students were continuously in contact with the teachers, teachers who may guide them to develop good habits throughout their life in school [30]. Thus, schools are a suitable place for educational interventions to increase knowledge, attitudes, and behavior for health promotion among adolescents. Therefore, this study aims to assess the effectiveness of an educational intervention program (interactive lecture) based on the theory of planned behavior for reducing junk food consumption among school adolescents in Birgunj Metropolitan City, Nepal. Findings of the study help to provide the tools for raising awareness, planning health promotion interventions, and promoting research to reduce junk food consumption behavior, especially among adolescents in a school setting. Moreover, the findings of the study will be contributing to a better handling of the practice of nutrition. Adolescents in the study setting were selected as no study has been done on this topic in this area.

## 2. Materials and Methods

Pretest and Posttest group designs were applied for conducting this study. Four out of twelve government schools of Birgunj Metropolitan City were undertaken for this study, from January 2018 to April 2018. These four schools were randomly selected, and from each school, complete enumeration was taken and students were invited to participate in the study. However, in this study, we only focused on the adolescent students of grade nine. While conducting the study, all the selected students were voluntarily asked for participation. Before recruiting them, they were fully informed about the study. Afterwards, parental consent was obtained with their signature/thumb print on the form and those adolescent students interested in attending the study also provided a written consent signed by them.

Furthermore, with the favourable information of the Ethics Committee of Pokhara University School of Health and Allied Sciences, study was conducted in two phases. In the first phase, we collected all the baseline information of junk food consumption by students involved for this study. This phase is also called the pretest. In the pretest, we used a structured questionnaire which was developed by intensive literature review based on the theory of planned behavior (TPB) [31, 32]. Then, we collected the data using a structured self-administered questionnaire which was prepared in Nepali language because the participants were not fully used to English; however, this questionnaire was initially prepared in English. Subsequently, from the four schools, 274 questionnaires were provided to students. Students of grade nine from each school were equally divided into 68, 68, 68, and 70, respectively. Then, they were asked to return them after completing it in the classrooms. In the second phase, we applied the intervention package (interactive lecture) for preventing the consumption of junk food to adolescents of grade nine students. The intervention package was given to same participants (274) in a similar setting of pretest during school hours. This activity was carried out with approval of head teacher and parents of participants. Therefore, this phase is also called as posttest. Moreover, tools used for this study consisted of five sections, namely, sociodemographic characteristics (age, gender, ethnicity, religion, father's occupation, father's education, mother's occupation, mother's education, and number of siblings), attitude about junk food use (most likely about junk food, taste, satisfaction, health, weight gain, and convenience), subjective norm (approval and influence on decision about what I eat: parent, teacher, friends, and siblings), perceived behavioral control (enabling factors: advertisement, cost/price, weight gain, easier to cook, limited time, enjoy the taste, and enjoy with friends and family), and behavioral intention (intend and plan to eat junk food). A 4-point Likert scale (1: very much, 2: somewhat, 3: a little, and 4: not at all) was applied to all the constructs of TPB. Afterwards, completed questionnaires were tested for validity and reliability, and it was found that Cronbach's alpha was 0.77, 0.83, 0.81, and 0.85 for behavior intention, attitude, subjective norm, and perceived behavior, respectively. Then pretesting was conducted on 10% of total sample size in similar setting but different school. Necessary changes were corrected accordingly after the pretesting of the tools. In this regard, this study aimed to assess the effectiveness of an educational intervention program (interactive lecture) based on the theory of planned behavior (TPB).

Moreover, the theory of planned behavior (TPB) assumes that human behaviors are built by behavioral intention affected by subjective norms, attitudes, and perceived behavioral control. In respect to junk food consumption, attitude could be personal or negative feelings, while “subjective norms” could be how much an individual likes to respect and pursue the opinions of a person who is important to him or her. “Perceived behavioral control” would be a person's perceived ability and external factors to use or disuse of junk food. In this study, pretest and posttest without a control group was carried out in three phases. In the first phase, we have done a pretest to identify the level of attitude, subjective norm, and perceived behavior control and behavior intention toward junk food consumption. In the second phase, the educational package was implemented. This package was interactive lectures of two sessions in school among the adolescent students, while in the third phase, a posttest was done. Meanwhile, the interventional package (educational intervention, i.e., interactive lecture) was developed after analyzing the knowledge, attitudes, behavior, and factors influencing the junk food consumption behavior of the participants through formative research. However, both the pretest and the posttest were done in the same study population. Ethical approval for the study was taken from the Institutional Review Committee (IRC) of Pokhara University, Nepal.

The data were entered into Epi Data 3.1 software. Then, they were exported to R for analysis of the data [33]. All the data files were checked and cleaned before analysis. Recoding was done to categorize the data and a composite score was calculated for attitude, subjective norms, perceived behavior control (PBC), and intention of participants. Afterwards, descriptive analyses including frequencies, mean, standard deviation, and range were performed. To analyze the hypothesis, paired *t*-tests were performed. Multiple linear regression model was computed to determine the relationship among the statement of attitude, perceived behavioral control, subjective norm, and intention to eat junk food.

## 3. Results and Discussion

**Figure 1:**
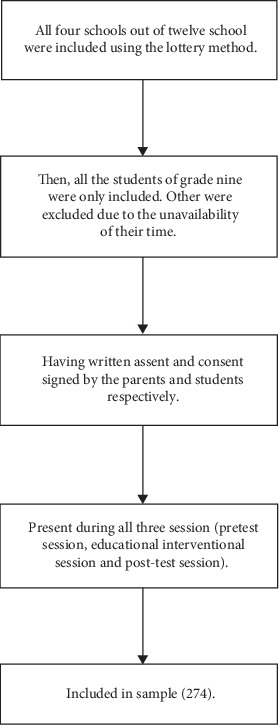
Flow diagram of inclusion of schools and adolescents.

All the students from grade nine of four government schools of Birgunj Metropolitan City were recruited in this study. Of these, most (40.1%) of the participants were disadvantaged non-Dalit caste of Terai region. More than two-thirds of the participants belonged to Hindu religion. After estimating the junk food consumption among the school-going adolescents from four different government schools of Birgunj, Figure 1 reflects the inclusion of adolescents and schools for this study.

### 3.1. Sociodemographic Characteristics

In total, 274 school adolescents were involved in the survey.  Table 1 shows the sociodemographic characteristics of respondents. Majority of the respondents were from middle adolescence (66.4%), were male students (59.9%), and were Hindu (74.8%). However, majority of their fathers (49.3%) and mothers (68.2%) were illiterate and most of them work in agriculture.

### 3.2. Behavioral Intention toward Junk Food Consumption

Students showed a statistically significant difference in intention in terms of their intention to eat (*P* < 0.0001) and plan to eat junk food over the next week (*P* < 0.0001) as shown in Table 2. The average score of behavioral intention toward junk food consumption during pretest was 5.43 ± 1.3, which was changed after the intervention 7.96 ± 0.3. With regard to the level of behavioral intention toward junk food consumption, most of the school-going adolescents changed their intention after the intervention. Before the intervention, 66.4% students had a high intention to consume junk food over the next week which was decreased to 1.8% after the intervention. The result of paired *t*-tests showed a statistically significant difference in behavioral intention toward junk food consumption after the intervention (*P* < 0.0001) as shown in Table 3.

### 3.3. Attitude toward Junk Food Consumption

Students' attitudes regarding taste (*P* < 0.0001), satisfaction (*P* < 0.0001), health (*P* < 0.0001), weight (*P* < 0.0001), and convenience (*P* < 0.0001) toward junk food showed a statistically significant difference after the intervention as shown in Table 2. The mean score of attitude toward junk food consumption during pretest was 11.9 ± 1.5, which was changed after the intervention to 16.3 ± 1.6. With regard to the level of attitude toward junk food consumption, most of the school-going adolescents developed a positive attitude after the intervention. Before the intervention, 28.8% of students had a positive attitude toward junk food per week, which was increased to 51.8% after the intervention. The result of paired *t*-tests showed a statistically significant difference in attitude toward junk food consumption after the intervention (<0.0001) as shown in Table 3.

### 3.4. Subjective Norm toward Junk Food Consumption

Subjective norm was assessed through parents' approvals, teachers' approvals, friends' approvals, and siblings' approvals for junk food consumption. Friends and siblings were highly influential on adolescents in school. The average rate of their friends' approval was 2.06 ± 0.80, which was significantly different after intervention, 3.22 ± 0.56 (*P* < 0.0001), and siblings' approval was 2.10 ± 0.68 which showed a statistically significant difference after intervention, 3.26 ± 0.57 (*P* < 0.0001), as shown in Table 2. A finding of the study showed that 53.3% of students before the intervention were positively influenced by subjective norm which was increased after the intervention to 66.1%. It was found that there was a statistically significant difference in subjective norm toward junk food consumption after the intervention (*P* < 0.0001) as shown in Table 3.

### 3.5. Perceived Behavior Control toward Junk Food

Perceived behavior control (BPC) was assessed through the influence of advertising, price, limited time, and easy accessibility of junk food in school. It was found that limited time had the greatest influence toward junk food. The average rate of limited time was 2.42 ± 0.50, which was significantly different after intervention, 3.13 ± 0.58 (*P* < 0.0001), and the influence of advertisements was 2.51 ± 0.52 which showed a statistically significant difference after intervention, 3.42 ± 0.53 (*P* < 0.0001), as shown in Table 2. It was found that intervention had a positive influence on the PBC of the students. More than half of the students were positively influenced by PBC after the intervention. It was also found that there was a statistically significant difference in PBC of junk food consumption after the intervention (*P* < 0.0001) as shown in Table 3.

Junk food is the more common and most preferable food in Nepal, especially with people under the age of 18 years. It was found that the majority (94%) of adolescents consume junk food. So, it is essential to consider how junk food preference is growing among the adolescents [24, 34]. Therefore, this study highlights the effectiveness of educational intervention based on theory of planned behavior (TPB) with the variables of attitude, subjective norms, perceived behavior, and behavioral intention toward junk food among the school-going adolescents by using the multiple linear regression model and paired *t*-test. Our study shows that 82.8% of adolescents had consumed junk food while they were in school. Of them, a higher percentage was clearly observed in the late adolescent period. This is possibly due to the preferred taste, the easy accessibility, and influence of advertisements, which has a major role to play in the temptation of having junk food. Similar studies also revealed that delicious taste, easily available, instantly eatable, and advertisement by the celebrities and sportsman are the key factors for consuming high proportion of junk food [9, 34, 35]. These findings indicate the serious health concerns among adolescents; if they consume it more frequently, it is often associated with negative health consequences [31]. Thus, these results of the study deliver the needed information regarding the effects of junk food among adolescents and help to encourage the implementation of appropriate rules and regulations related to lowering the consumption of junk food in homes and also in school. In this study, it was found that the intention of consuming junk food was decreased by the attitude, subjective norm, and perceived behavioral control. This is primarily due to the considerable investment in the education intervention package which brought about the negative attitude toward the consumption of junk food. Other studies conducted in different parts of the world suggested that if the people received the knowledge from any source of media/methods, then their intention was changed by health education activities and thus reduced the intake of junk food [36–38]. Therefore, students will become less inclined to eat junk food in the case where they have changed attitudes toward junk food, and their intentions, or pressures on them, to use junk food are low. Thus, adolescent positive attitudes should be reinforced in practice through the educational interventions.

Furthermore, the mean score of behavioral intention for reducing junk food consumption was increased after the intervention. The mean score of behavioral intention during pretest was 8.2 while 11.9 during posttest. Increase in the negative attitude toward the junk food consumption has reasonably assumed that there is an increase in the level of knowledge on junk food which helped to increase the level of awareness and prevent the high consumption of junk food. Similar findings of our study were matched with a few other studies which showed that education is the key strategy to bring positive changes toward junk food consumption. In addition, after changing their awareness level, the overall mean score was found as 3.93 in pretest, while it was 5.34 in posttest [39].

Moreover, this study was also measuring the effectiveness of the educational intervention toward the attitude, subjective norm, PBC, and behavioral intention. It was found that all the constructed variables were significantly (<0.0001) associated with behavioral intention. Also, their mean differences (attitude [9 ± 1.5, 16.3 ± 1.6], subjective norms [11.1 ± 1.3, 14.3 ± 1.4], PBC [9.9 ± 1.0, 12.76 ± 1.5], and BI [5.43 ± 1.3, 7.96 ± 0.3]) were also found to have significant differences in pretest and posttest. Educational intervention was significant with attitude (*t* = 32.62, *P* < 0.001), subjective norm (*t* = 52.92, *P*=0.001), and PBC (*t* = 23.07, *P* < 0.001). Additionally, significant difference in behavioral intention for the prevention of junk food consumption after educational interventions is also found in other studies (*t* = 30.6, *P* < 0.001) [40]. Consequently, it was found that the educational intervention had the major role in preventing the consumption of junk food. These strategies act to increase the positive thinking felt by adolescents and lead to developing the positive effect of health and development [39]. Hence, this study indicated that junk food consumption was predominantly affected by the education interventional package, and, therefore, the school authorities and government should pay more attention toward junk food and provide needed packages against the consumption of junk food in schools.

## 4. Conclusions

This study shows the effectiveness of an educational intervention program among the school-going adolescent students using the TPB model. It was found that the interactive lecture method was an effective technique for changing the intention to consume junk food. Hence, the educational intervention program is effective for changing the student's attitude, subjective norm, and perception behavior toward the consumption of junk food. Additionally, the findings of the study reflect that TPB is effective for interventional programs for enhancing positive behaviors. Thus, it is suggested that similar studies need to be performed in other communities and districts, at the regional and national levels in Nepal.

### 4.1. Limitations of the Study

Our study had some limitations. Firstly, the study focused only the ninth-grade students from selected schools; we did not include the grade eight and grade ten students due to unavailability of their time. Secondly, we cannot explore the behavior on junk food among the school-going adolescents due to the short period of time. So, it may not be possible to evaluate the effectiveness of intervention for preventing the consumption of junk food.

## Figures and Tables

**Table 1:** Sociodemographic characteristics.

Variables		Frequency	Percent
Age	Middle adolescents	182	66.4
	Late adolescents	92	35.6

Sex	Male	164	59.9
	Female	110	40.1

Religion	Hindu	205	74.8
	Muslim	43	15.7
	Buddhist	15	5.5
	Christian	11	4

Father's education	Illiterate	135	49.3
	Primary	10	3.6
	Lower secondary	21	7.7
	Secondary	61	22.3
	Higher secondary and above	47	17.1

Mother's education	Illiterate	187	68.2
	Primary	26	9.5
	Lower secondary	23	8.4
	Secondary	21	7.7
	Higher secondary and above	17	6.2

Father's occupation	Government employee	25	9.1
	Business	38	13.9
	Private service	57	20.8
	Agriculture	100	36.5
	Foreign employee	30	10.9
	Others	24	8.8

Mother's occupation	Government employee	1	0.4
	Business	33	12
	Private service	28	10.2
	Agriculture	83	30.3
	Housewife	125	45.6
	Others	4	1.5

**Table 2:** Constructs of theory of planned behavior toward junk food consumption.

Statements	Pretest Mean ± SD	*P* value	Posttest Mean ± SD	*P* value
Attitude toward junk food				
I like taste of junk food	2.23 ± 0.77	**<0.0001**	3.48 ± 0.61	**<0.0001**
Satisfaction after eating junk food	2.01 ± 0.61	**<0.0001**	3.02 ± 0.43	**<0.0001**
Junk food is good for health	2.75 ± 0.46	**<0.0001**	3.90 ± 0.29	**<0.0001**
Junk food increases the weight	2.43 ± 0.53	**<0.0001**	2.94 ± 0.51	**<0.0001**
It is convenient to prepare junk food	2.49 ± 0.54	**<0.0001**	3.00 ± 0.47	**<0.0001**
Subjective norm toward junk food				
Parents' approval for junk food	2.94 ± 0.24	<0.0001	3.90 ± 0.28	**<0.0001**
Teachers' approval for junk food	3.93 ± 0.32	<0.0001	3.93 ± 0.25	**<0.0001**
Friends' approval for junk food	2.06 ± 0.80	<0.0001	3.22 ± 0.56	**<0.0001**
Siblings' approval for junk food	2.10 ± 0.68	<0.0001	3.26 ± 0.57	**<0.0001**
Perceived behavioral control for junk food				
Advertisement influences me to eat junk food	2.51 ± 0.52	<0.0001	3.42 ± 0.53	**<0.0001**
Price influences me to eat junk food	2.48 ± 0.50	<0.0001	2.99 ± 0.44	**<0.0001**
Limited time influences me to eat junk food	2.42 ± 0.50	<0.0001	3.13 ± 0.58	**<0.0001**
Easy accessibility of junk food in school influences me to eat junk food	2.48 ± 0.52	<0.0001	3.21 ± 0.55	**<0.0001**
Behavioral intention toward junk food				
I intend to eat junk food over the next week	2.71 ± 0.74	<0.0001	3.98 ± 0.13	**<0.0001**
I plan to eat junk food over the next week	2.71 ± 0.74	<0.0001	3.98 ± 0.13	**<0.0001**

**Table 3:** Level of construct before and after intervention and comparing the mean score of construct of TPB before and after intervention.

Construct of TPB					
	Pretest *n* (%)	Posttest *n* (%)	Pre *t*-test Mean CSD	Post *t*-test Mean ± SD	*P* value
Attitude			11.9 ± 1.5	16.3 ± 1.6	<0.0001
Positive (≤mean)	79 (28.8%)	142 (51.8%)			
Negative (>mean)	195 (71.2%)	132 (48.2%)			
Subjective norm			**11.1** **±** **1.3**	**14.3** **±** **1.4**	**<0.0001**
Positive (≤mean)	146 (53.3%)	181 (66.1%)			
Negative (>mean)	128 (46.7%)	93 (33.9%)			
Perceived behavioral control			9.9 ± 1.0	12.76 ± 1.5	**<0.0001**
Positive (≤mean)	105 (38.3%)	142 (51.8%)			
Negative (>mean)	169 (61.7%)	132 (48.2%)			
Behavioral intention			**5.43** **±** **1.3**	**7.96** **±** **.3**	**<0.0001**
High (≤mean)	182 (66.4%)	5 (1.8%)			
Low (>mean)	92 (33.6%)	269 (98.2%)			

## Data Availability

Data will be provided upon reasonable request from the corresponding author.
